# Supplementary material for: The Potential Role of Complement System in the Progression of Ovarian Clear Cell Carcinoma Inferred from the Gene Ontology-Based Immunofunctionome Analysis
Source: Int J Mol Sci. 2020 Apr 17;21(8):2824. doi: 10.3390/ijms21082824 (PMC7216156; doi:10.3390/ijms21082824)
Supplement: Supplementary file 1 [file ijms-21-02824-s001.zip › ijms-730600-Supplementary Files-to conversion/Figure S1 Three immune-related genes (CFP, C9, and C5) of the complement system associated with good survival outcomes.pdf]

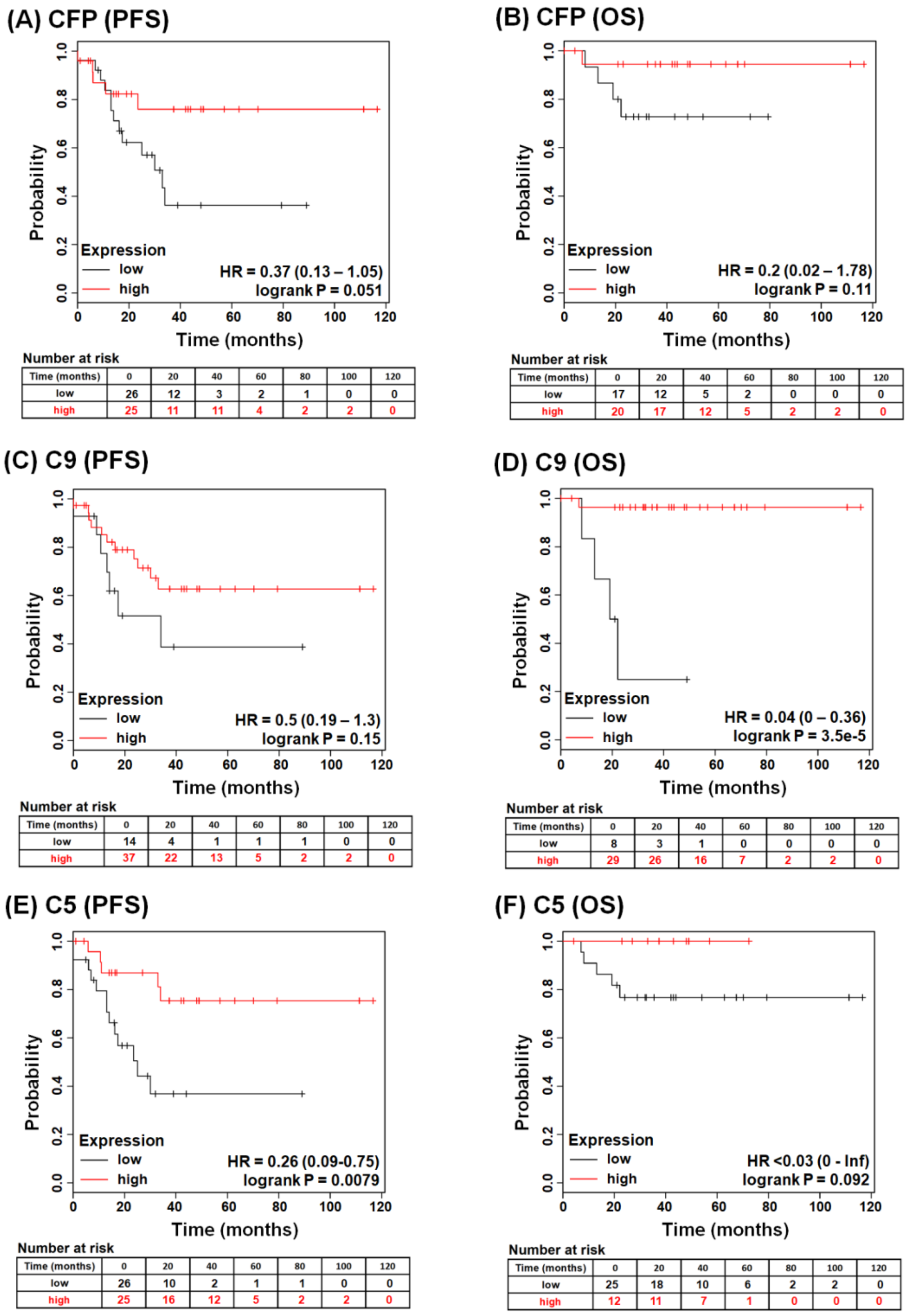

Figure S1. Three immune-related genes (CFP, C9, and C5) of the complement system associated with good survival outcomes (progression-free survival (PFS, (A), (C), (E)) and overall survival (OS, (B), (D), (F))) in EAOC.

The hazard ratios of the PFS of CFP, C9, C5 were 0.37(0.13-1.05, p = 0.051), 0.5(0.19-1.3, p = 0.15), 0.26(0.09-0.75, p = 0.0079), and the hazard ratios of the OS of CFP, C9, C5 were 0.2(0.02-1.78, p = 0.11), 0.04(0-0.36, p = 3.5 × 10-5), <0.03(0-inf, p = 0.092), respectively.
