# Supplementary material for: The Potential Role of Complement System in the Progression of Ovarian Clear Cell Carcinoma Inferred from the Gene Ontology-Based Immunofunctionome Analysis
Source: Int J Mol Sci. 2020 Apr 17;21(8):2824. doi: 10.3390/ijms21082824 (PMC7216156; doi:10.3390/ijms21082824)
Supplement: Supplementary file 1 [file ijms-21-02824-s001.zip › ijms-730600-Supplementary Files-to conversion/Figure S2 Four immune-related genes (VSIG, C8b, C7 and C5) of the complement system associated with poor survival outcomes.pdf]

(A) VSIG4 (PFS)

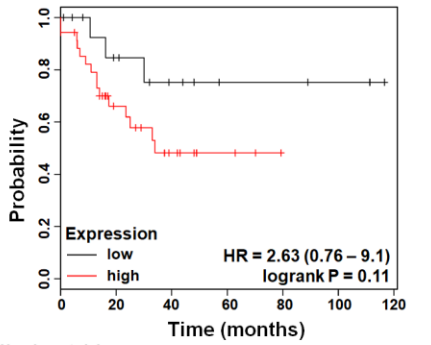

Number at risk

Time (months)	0	20	40	60	80	100	120
low	16	10	6	3	3	2	0
high	35	16	8	3	0	0	0

(B) VSIG4 (OS)

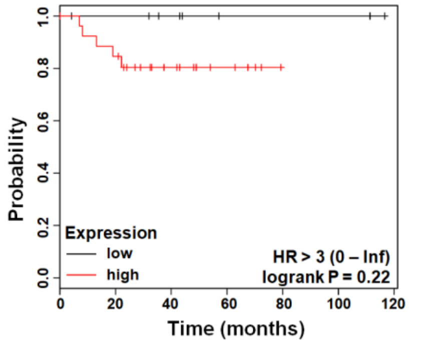

Number at risk

Time (months)	0	20	40	60	80	100	120
low	8	7	5	2	2	2	0
high	29	22	12	5	0	0	0

(C) C8B (PFS)

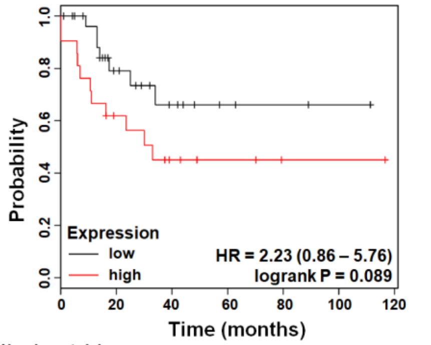

Number at risk

Time (months)	0	20	40	60	80	100	120
low	30	15	8	3	2	1	0
high	21	11	6	3	1	1	0

(D) C8B (OS)

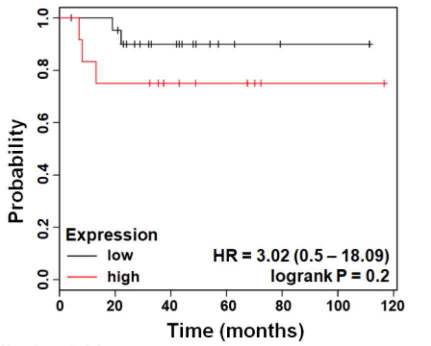

Number at risk

Time (months)	0	20	40	60	80	100	120
low	25	20	11	3	1	1	0
high	12	9	6	4	1	1	0

(E) C7 (PFS)

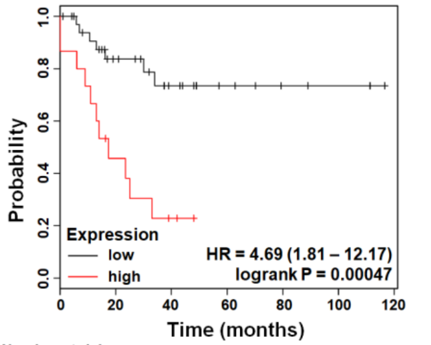

Number at risk

Time (months)	0	20	40	60	80	100	120
low	36	20	12	6	3	2	0
high	15	6	2	0	0	0	0

(F) C7 (OS)

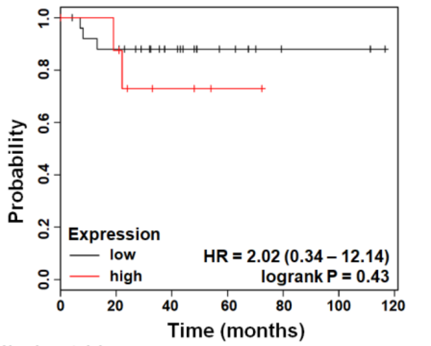

Number at risk

Time (months)	0	20	40	60	80	100	120
low	27	22	14	6	2	2	0
high	10	7	3	1	0	0	0

(G) C3 (PFS)

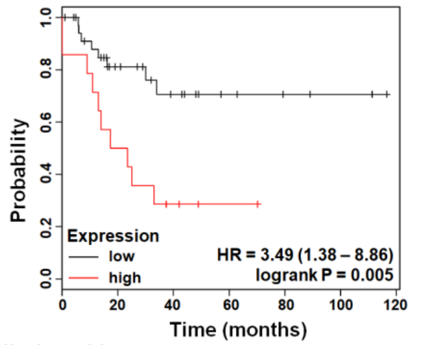

Number at risk

Time (months)	0	20	40	60	80	100	120
low	37	19	11	5	3	2	0
high	14	7	3	1	0	0	0

(H) C3 (OS)

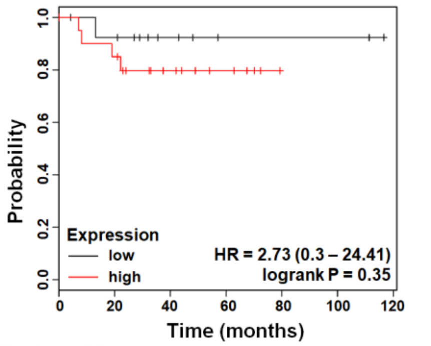

Number at risk

Time (months)	0	20	40	60	80	100	120
low	15	12	7	2	2	2	0
high	22	17	10	5	0	0	0

Figure S2. Four immune-related genes (VSIG, C8b, C7 and C5) of the complement system associated with poor survival outcomes (progression-free survival (PFS, (A), (C), (E), (G)) and overall survival (OS, (B), (D), (F), (H))) in EAOC.

The hazard ratios of the PFS of VSIG4, C8B, C7, C3 were 2.63(0.76-9.1, p = 0.11), 2.23(0.86-5.76, p = 0.089), 4.69(1.81-12.17, p = 0.00047), 3.49(1.38-8.86, p = 0.005), and the hazard ratios of the OS of VSIG4, C8B, C7, C3 were >3(0-inf, p = 0.22), 3.02(0.5-18.9, p = 0.2), 2.02(0.34-12.14, p = 0.43), 2.73(0.3-24.41, p = 0.35), respectively.
